# Twenty-Four-Hour Urinary Sodium Excretion Predicts Therapeutic Effectiveness of Oral Rehydration Saline in Pediatric Vasovagal Syncope

**DOI:** 10.3390/children9070992

**Published:** 2022-07-01

**Authors:** Xiaojuan Du, Chunyan Tao, Yaru Wang, Yan Sun, Qingyou Zhang, Chunyu Zhang, Ping Liu, Yuli Wang, Ying Liao, Junbao Du, Hongfang Jin

**Affiliations:** 1 Department of Pediatrics, Peking University First Hospital, Beijing 100034, China; 18801238002@163.com (X.D.); TaoCY126@126.com (C.T.); yaruw123@163.com (Y.W.); sunyan2007bjmu@163.com (Y.S.); zhangqingyou_73@126.com (Q.Z.); chunyu5781@163.com (C.Z.); duoduomama19731013@126.com (P.L.); mengyu1129@126.com (Y.W.); junbaodu1@126.com (J.D.); 2 Key Laboratory of Molecular Cardiovascular Sciences, Ministry of Education, Beijing 100191, China

**Keywords:** urinary sodium excretion, vasovagal syncope, oral rehydration saline, children, head-up tilt test

## Abstract

The study was designed to explore whether 24-hour urinary sodium excretion could predict the therapeutic effectiveness of oral rehydration saline in pediatric cases of vasovagal syncope. Eighty children suffering from vasovagal syncope with oral rehydration saline treatment in Department of Pediatrics, Peking University First Hospital, China, were recruited into the study. They were followed up for 3 (2, 3) months after treatment. Pre-treatment demographic, clinical, head-up tilt test-based hemodynamic and laboratory variables were compared between responders and non-responders. After univariate analysis, variables with *p* value < 0.05 in the comparison between responders and non-responders were further analyzed by binary logistic regression analysis. Receiver operating characteristic (ROC) curve was conducted to assess the value in predicting effectiveness of oral rehydration saline treatment. The results showed that 33 cases were responders, and 47 were non-responders. Blood sodium (138 ± 2 mmol/L vs. 139 ± 2 mmol/L, *p* < 0.05) and pre-treatment 24-hour urinary sodium excretion (74 ± 29 mmol/24 h vs. 109 (93, 141) mmol/24 h, *p* < 0.001) were lower in responders than in non-responders. The baseline 24-hour urinary sodium excretion was positively correlated to the duration from tilting to the positive response appearance in head-up tilt test (r = 0.289, *p* < 0.01). The cut-off value of baseline 24-hour urinary sodium excretion of the therapeutic effectiveness of oral rehydration saline on vasovagal syncope cases was 83 mmol/24 h, yielding a sensitivity of 87% and a specificity of 73% with AUC of 0.842 (*p* < 0.001). In conclusion, 24-hour urinary sodium excretion could be a useful biomarker to predict the therapeutic response to oral rehydration saline in pediatric cases of vasovagal syncope.

## 1. Introduction

Vasovagal syncope is a prevalent disease entity of syncope in children, accounting for more than 60% of all pediatric fainting cases [1]. Oral rehydration saline is a common treatment, which mainly supplements the sodium salt and fluid to the patients to maintain a more stable circulating blood volume, but the efficacy of the unselected use of oral rehydration saline treatment in vasovagal syncope is not satisfactory [2,3], which might be related to the complexity and diversity of the pathogenesis of vasovagal syncope.

At present, it is believed that the pathogenesis of vasovagal syncope mainly includes low circulatory blood volume [4,5], high catecholamine status [6,7], excessive vasorelaxation [8], etc. Considering the central role of sodium in determining fluid balance, circulating volume and blood pressure [9], it appears clear why oral rehydration saline is efficacious only for those with low salt and low circulatory blood volume, since it increases the volume of circulating blood flow. Therefore, to accurately identify the vasovagal syncope children with low salt and low circulatory blood volume as the main pathogenesis before treatment and thereby provide the target treatment for children with oral rehydration, saline is an important clinical issue and merits studies.

Body mass index (BMI) is a classic and easy-to-obtain measure which is correlated with the body blood volume [4,10]. It has been used as a predictor of the outcome of oral rehydration saline treatment in pediatric cases of vasovagal syncope. Sodium excretion might be a further pathophysiologically based biomarker to predict the outcomes of vasovagal syncope children treated with oral rehydration saline. Indeed, it has been reported that 24 h sodium urine reflected the body reserves of sodium ions to some extent, reflecting severity of vasovagal syncope [11]. This study, therefore, aimed to examine whether 24-hour urinary sodium excretion could predict the therapeutic response to oral rehydration saline in pediatric cases of vasovagal syncope.

## 2. Materials and Methods

### 2.1. Study Subjects

This is a case-series study. The study included 84 vasovagal syncope patients who visited the pediatric department, Peking University First Hospital, China and received oral rehydration saline treatment from August 2012 to June 2021. Their median age was 10 years [8,9,10,11,12,13]. The comprehensive medical history, findings on physical examinations, and the results of the laboratory examinations such as blood biochemical examinations, electrocardiograms, dynamic electrocardiograms, echocardiography, and electroencephalograms were recorded from the Medical Recording Management Digital System (Kaihua, Beijing, China) to rule out cardiovascular or nervous system illness.

Based on the Chinese guidelines [12], vasovagal syncope is diagnosed with the following criteria: (1) fainting is often precipitated by susceptible triggers, such as posture from supine to upright, long-term upright posture and sultry environment; (2) having fainting attack; (3) positive response in head-up tilt test; and (4) excluding other causes of fainting-like events, such as epilepsy, hypoglycemia, and cardiac syncope. The study complied with the Declaration of Helsinki and the permission of Institutional Ethics Committees of Peking University First Hospital (N0.2021690).

Inclusion criteria were: (1) pediatric patients aged 5–16 years old hospitalized at Peking University First Hospital; (2) children diagnosed with vasovagal syncope; (3) with oral rehydration salt treatment; and (4) with demographic data, complete clinical data, electrocardiogram, head-up tilt test data and 24 h urine sodium data.

Exclusion criteria were: (1) patients with unknown diagnosis or diagnosis of vasovagal syncope combined with postural tachycardia syndrome; (2) patients with cardiac syncope or pseudosyncope; (3) patients with vomiting, diarrhea, administration of antiemetic, laxatives and diuretics; (4) oral rehydration saline treatment time less than 1 month [12]; and (5) incomplete data.

### 2.2. Head-Up Tilt Test

Head-up tilt test program and positive tests were consistent with the previous description [13,14]. The tests were carried out in the morning in dimly lit, warm and quiet rooms. Fasting was needed for at least 4 h and drugs that affect autonomic nerve function were forbidden for at a minimum 5 half-lives prior to head-up tilt test. Children laid on an upright tilt bed for 10–20 min to monitor the baseline physiological indicators. The tilt bed was positioned at 60° until a positive test occurred, or if no positive test occurred, the entire course of 45 min was completed. Positive response is as described previously [12]. Each patient’s legal guardian had signed the head-up tilt test informed consent.

### 2.3. Urine Test

A standard urine collection process was offered to each patient, who was asked to collect all urine for 24 h except the first urine. For females, 24 h urine was obtained outside the menstrual period. The 24-hour urinary sodium excretion was tested in an automatic chemical analyzer using standard biochemical detection methods, as described previously [15,16].

### 2.4. Therapy and Follow-Up

**Figure 1:**
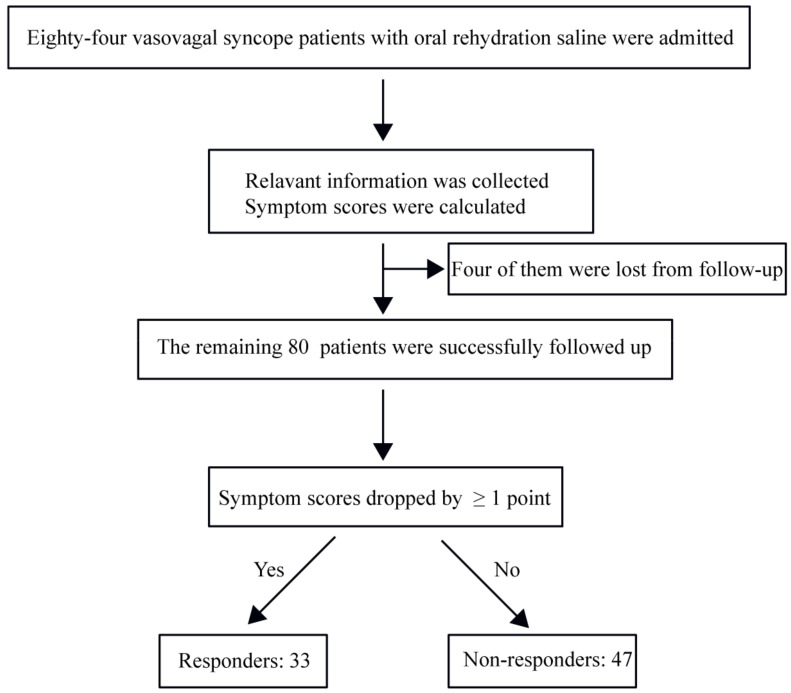
Flowchart of the study subject recruitment.

All enrolled children were treated with oral rehydration saline. After three months of sustained treatment, patients were followed up by a professionally trained researcher at the outpatient department of Peking University First Hospital, China or over telephone. None of the patients experienced adverse reactions to saline treatment, such as hypertension. For each patient, a syncope symptom score was assessed both before and after the treatment and those with a score of ≥1 were included in the study as described previously [4]. Treatment was defined as effective and the patient was termed responder (Figure 1) when the post-treatment score was at least 1 point lower than the pre-treatment score [17].

### 2.5. Statistics

Data were analyzed with SPSS 26.0 (IBM, Armonk, NY, USA). The normality of distribution of continuous variables was measured by the Kolmogorov–Smirnov test. Normal distribution data were presented as mean and standard deviation, and analyzed by Student *t*-tests. If the data were not normally distributed, they were presented as median and interquartile range, and analyzed by the Mann–Whitney U test. The stepwise logistic regression was executed to test the associated variables with the therapeutic response to oral rehydration saline. To evaluate the correlation between two variables, we used the Spearman test. The predictive value was defined by Youden indices using receiver operating characteristic (ROC) curve. The predictive ability was measured using the area under the curve (AUC). *p* < 0.05 denoted statistical significance.

## 3. Results

### 3.1. Baseline Characteristics

Eighty-four vasovagal syncope patients treated with oral rehydration saline were admitted to the study, four of whom (4.8%) were lost from follow-up (Figure 1). The remaining 80 patients were enrolled. The median duration of treatment was 3 (2.0, 3.0) months. According to whether the symptoms were relieved after treatment, 33 patients were classified as responders, and 47 patients as non-responders. No meaningful differences were found between responders and non-responders in sex, age, BMI, urine specific gravity, treatment time, symptom scores before treatment and head-up tilt test hemodynamic characteristics (*p* > 0.05). However, blood sodium, pre-treatment 24-hour urinary sodium excretion and symptom scores after treatment were lower in responders than in non-responders (*p* < 0.05) (Table 1).

### 3.2. Associated Variables with the Effect of Oral Rehydration Saline Treatment

The two significant baseline variables obtained from the univariate analysis in the comparison between responders and non-responders (blood sodium and 24-h sodium excretion) were further analyzed by binary logistic regression. The result showed that the twenty-four-hour urine sodium excretion was found to be associated with therapeutic response to oral rehydration saline (OR = 0.957, 95% CI = 0.937–0.978, *p* < 0.05).

### 3.3. Twenty-Four-Hour Urine Sodium Excretion Was Correlated with Vasovagal Syncope Severity

**Figure 2:**
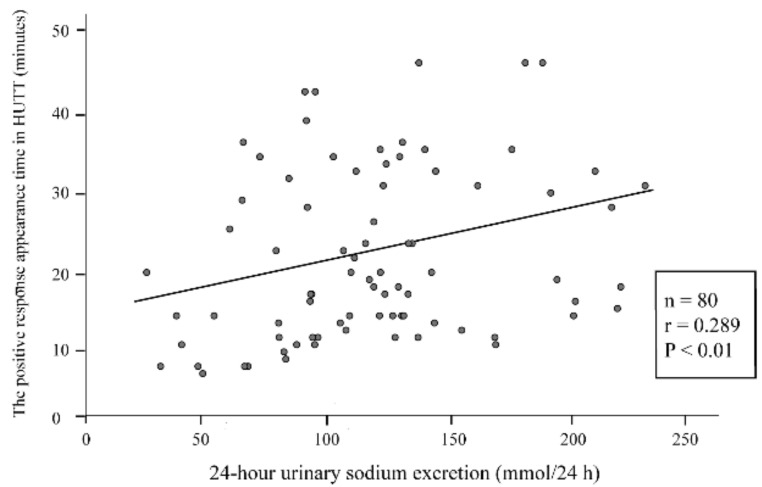
Correlation between 24-hour urinary sodium excretion and the duration from tilting to positive response appearance in head-up tilt test (HUTT).

Spearman’s correlation analysis was performed to evaluate the correlation between 24-hour urinary sodium excretion and the severity of the vasovagal syncope. The results were positively correlated between 24-hour urinary sodium excretion and head-up tilt test response time (r = 0.289, *p* < 0.01) (Figure 2).

### 3.4. Baseline Twenty-Four-Hour Urine Sodium Predicted the Therapeutic Response to Oral Rehydration Saline

**Figure 3:**
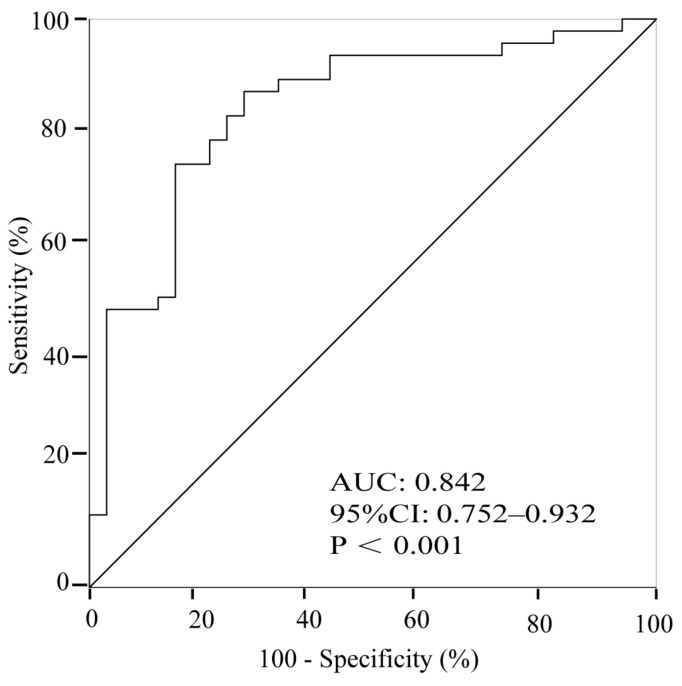
The ROC curve of 24-hour urinary sodium excretion to predict the response to oral rehydration saline treatment in pediatric cases of vasovagal syncope.

ROC curve analysis showed that baseline 24 h urine sodium excretion had high accuracy in predicting the therapeutic response to oral rehydration saline with an AUC of 0.842 (95% CI: 0.752 to 0.932, *p* < 0.001) (Figure 3). The cut-off value of 24 h urine sodium excretion was 83 mmol/24 h, yielding a sensitivity of 87% and a specificity of 73%, respectively.

## 4. Discussion

Our study revealed that baseline 24-hour urinary sodium excretion was notably lower in responders than in non-responders. The baseline 24-hour urinary sodium excretion less than 83 mmol/24 h could predict the response to oral rehydration saline treatment in pediatric cases of vasovagal syncope.

Many medical centers use salt supplements as the first-line treatment of vasovagal syncope [3,18], but only less than 50% of patients treated with oral rehydration saline show satisfactorily clinical response [3], and the changing rate from positive to negative response to head-up tilt test was only 48.2% [2]. The reason may be the fact that the pathogenesis of vasovagal syncope is complex and diverse, including low circulating blood volume, high catecholamine state, excessive vasodilation, etc. [19,20,21,22], while as a matter of fact, oral rehydration saline would only be effective for vasovagal syncope patients with low blood volume. When standing up, a certain volume of blood will be transferred from the blood vessels of the head and upper limbs to those of the lower limbs and the internal organs, resulting in a reduction in blood flow back to the heart. For vasovagal syncope patients with low blood volume, the amount of blood returned to the heart is decreased significantly when they stand up. Due to the Frank–Starling rule, this leads to a significant decrease in cardiac output and subsequently in cerebral blood flow, and syncope may occur.

The main components of oral rehydration saline are water and sodium salt. As we know, the amount of sodium in the body impacts the amount of extracellular fluid (including plasma) [23]. Supplementing water and salt would have an “anti-gravity” effect on the pre-syncope, enhance the blood volume changes under the background of changes in body position and increase the tolerability to avoid the occurrence of syncope. It has been supported by randomized controlled trials as well as meta-analysis that children with vasovagal syncope can benefit from the oral rehydration saline therapy, which can provide a quantified dosage of supplemental water and salt on the basis of the patients’ original diet [3,18]. In addition to the supplementary sodium chloride and fluid volume, other components of oral rehydration saline, such as glucose, can promote the absorption of sodium ions at the small intestine, which may enhance the efficacy of oral rehydration saline [24,25]. Therefore, looking for indicators that can reflect the decrease in blood volume to suggest the oral rehydration saline treatment to the vasovagal syncope patients with low circulating blood volume as the pathogenesis is interesting and potentially useful. This pathophysiological role is the main interest of 24 h urine sodium, as opposed to BMI which is easier to obtain in daily clinical life.

Indeed, sensitivity and specificity of BMI (sensitivity 83%, specificity 73%) were similar [4] to those obtained for 24-hour urinary sodium excretion in the current study. However, BMI was not different between the responders and non-responders in our study. One possible reason may be that the proportion of responders in the total population is different between the present study (33/80) and the previous study [4]. The small sample size of both studies should also be taken into account.

In this study, we showed that the 24-hour urinary sodium excretion was positively correlated to head-up tilt test response time of children with vasovagal syncope. However, the data are scattered underscoring the big interindividual variability, and the r value is low (r = 0.289), which may also be affected by the relatively small sample size. We also showed that the responders had significantly lower baseline 24 h urine sodium excretion than the non-responders. In particular, patients with 24-hour urinary sodium excretion lower than 83 mmol/24 h before treatment responded well to oral rehydration saline, with a predictive sensitivity of 87% and a specificity of 73%, helping in offering an individualized treatment to the affected patients.

There are, of course, some limitations in our study. Firstly, the short follow-up period, small sample size and potential selection bias intrinsic to study design may lead to biased results. Secondly, this study assessed each patient’s 24-hour urinary sodium excretion only once, and daily changes are also possible. In future trials, 24-hour urinary sodium excretion should be repeatedly assessed in post-treatment patients. Thirdly, our study only included those treated with oral rehydration saline alone, which, as a basic non-pharmacological treatment, may often be used by physicians in children with milder conditions, leaving our study with some selection bias that needs to be further confirmed in future prospective studies. In addition, in an effort to standardize fluid and salt intake, in this preliminary study, we tested only commercial oral rehydration solutions. However, the simple advice of generous drinking and eating salty is the most widespread management strategy for vasovagal syncope. This should be investigated in further studies. In conclusion, despite the present limitations, this study showed that 24-hour urinary sodium excretion is a valuable indicator for predicting the response to oral rehydration saline treatment in pediatric cases of vasovagal syncope potentially providing some basis for an individualized treatment of vasovagal syncope. Large sample-sized and sufficiently powered studies are warranted to confirm these results.

## Figures and Tables

**Table 1:** Comparison of demographic, clinical and laboratory results between responders and non-responders.

Characteristics	Treatment Response		t/Z/χ2 Value	*p*-Value
	Responders (*n* = 33)	Non-Responders (*n* = 47)		
Sex (female/male, *n*)	17/16	28/19	0.963	0.326
Age at head-up tilt test (years)	10 ± 2	11 (9, 13)	−1.590	0.112
Baseline body mass index (kg/m^2^)	16 (15, 19)	17 (16, 19)	−1.046	0.296
Duration of treatment (months)	3 (2, 3]	3 (2, 3)	−0.431	0.666
Symptom score before treatment (points)	1 (1, 2]	1 (1, 2)	0.553	0.581
Symptom score at follow-up (points)	0 (0, 0]	1 (1, 2)	−7.796	<0.0001
Supine heart rate (bpm)	75 (71, 88)	75 ± 11	1.056	0.291
Systolic blood pressure (mmHg)	105 ± 10	104 ± 8	0.184	0.854
Diastolic blood pressure (mmHg)	63 ± 8	61 ± 7	1.544	0.127
Response time in head-up tilt test (minutes)	13 (7, 28)	16 (10, 28)	−1.311	0.190
Blood sodium (mmol/L)	138 ± 2	139 ± 2	−2.067	0.042
Urine specific gravity	1.02 ± 0.01	1.02 ± 0.01	−0.147	0.884
24 h urine output (mL)	1262 ± 488	1370 ± 631	−0.826	0.411
24-hour urinary sodium excretion (mmol/24 h)	74 ± 29	109 (93, 141)	−5.185	<0.0001

## Data Availability

The data presented in this study are available on request from the corresponding author.
